# Supplementary material for: Synthesis, Characterization and Biological Activities of Zinc Oxide Nanoparticles Derived from Secondary Metabolites of Lentinula edodes
Source: Molecules. 2023 Apr 17;28(8):3532. doi: 10.3390/molecules28083532 (PMC10143259; doi:10.3390/molecules28083532)
Supplement: Supplementary file 1 [file molecules-28-03532-s001.zip › molecules-2297165-supplementary.pdf]

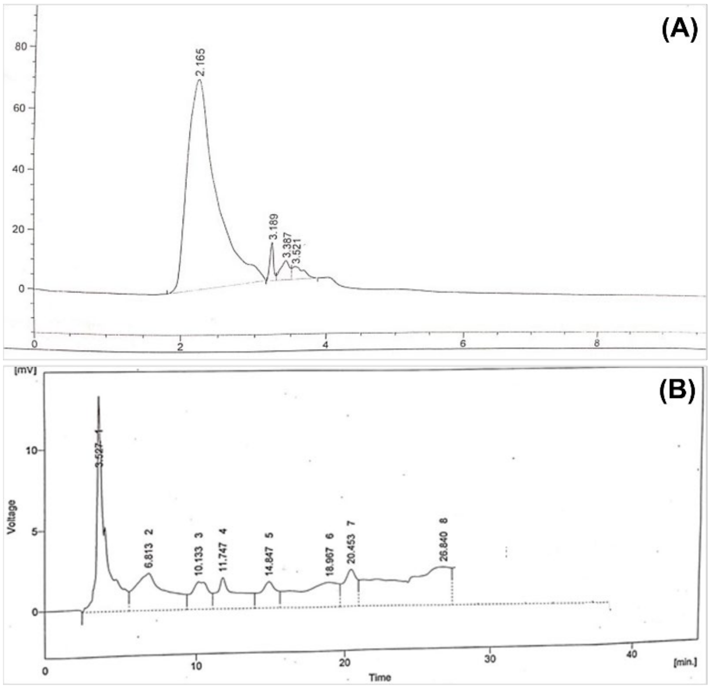

Figure S1. HPLC chromatograms of aqueous fraction of methanolic extract of Lentinula edodes (a) flavonoids (b) Phenolic acids.

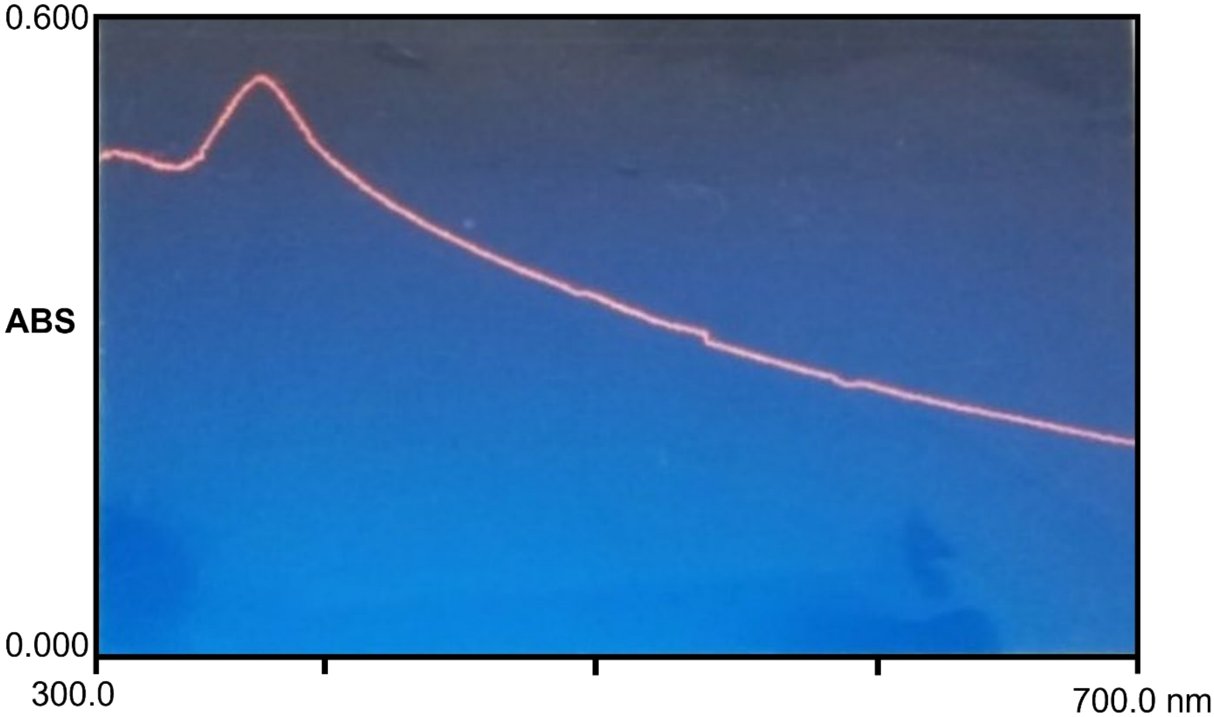

Figure S2. UV-Vis spectrum of ZnO NPs.

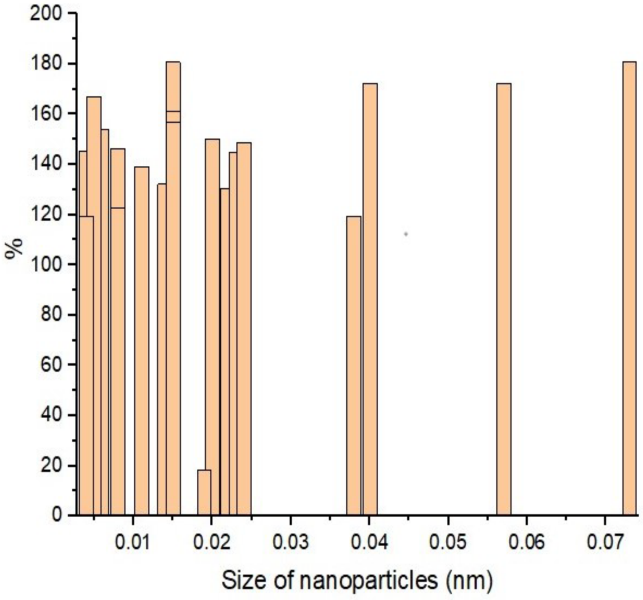

Figure S3. Size distribution of nanoparticles (SEM).

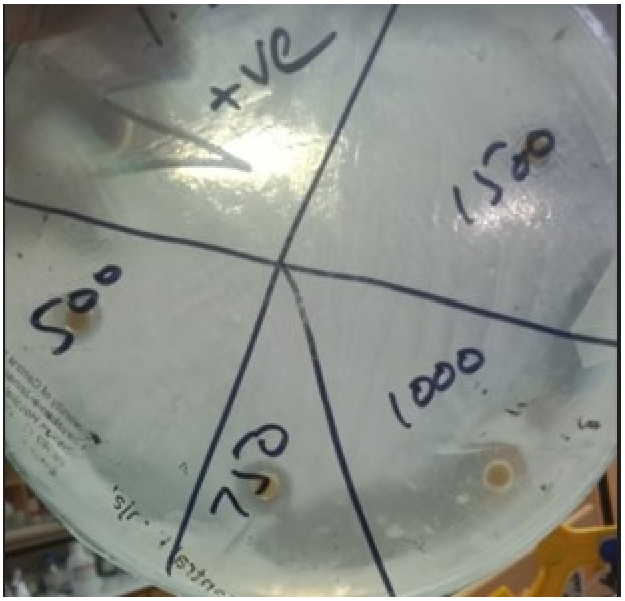

Figure S4. Inhibition zone of ZnO nanoparticles against *Escherichia coli*.

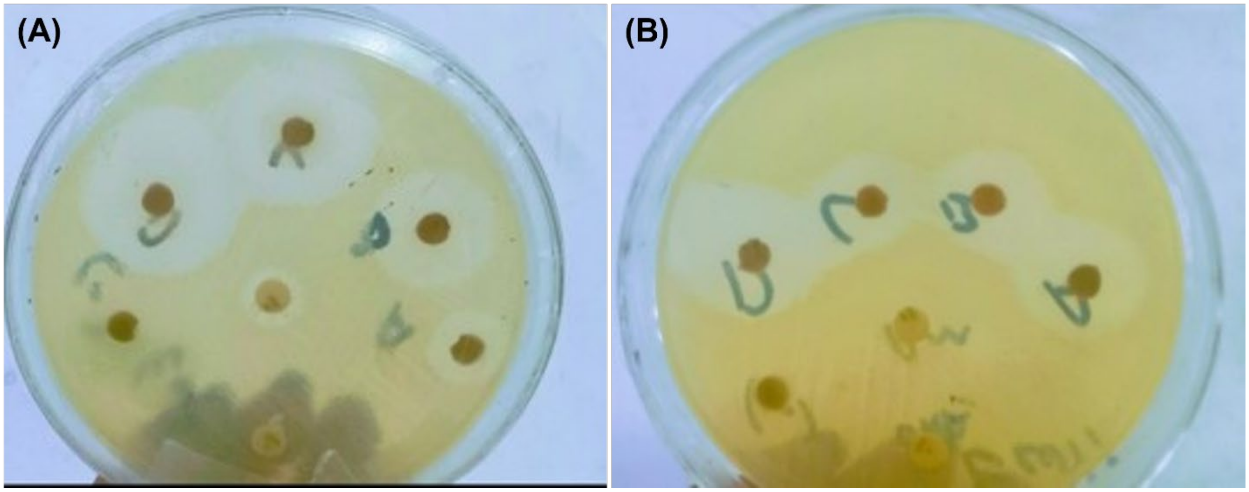

Figure S5. Inhibition zone of ZnO nanoparticles against (a) Staphylococcus aureus and (b) Klebsiella.

In vivo Antipyretic and Anti-inflammatory Activity

In vivo Anti- inflammatory Activity

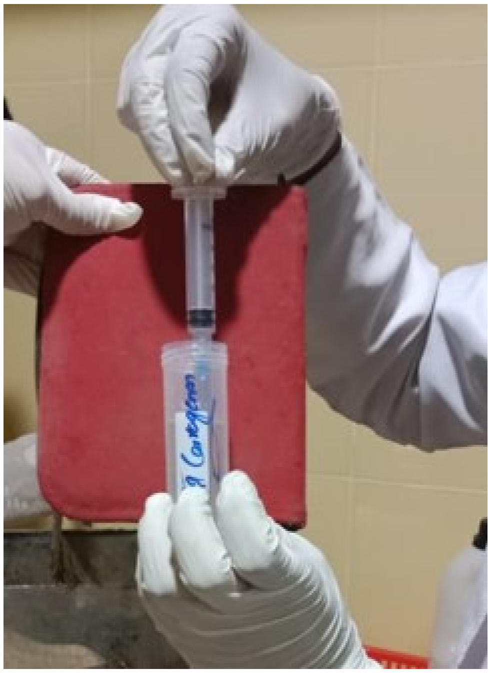

Figure S6. 1% Carrageenan.

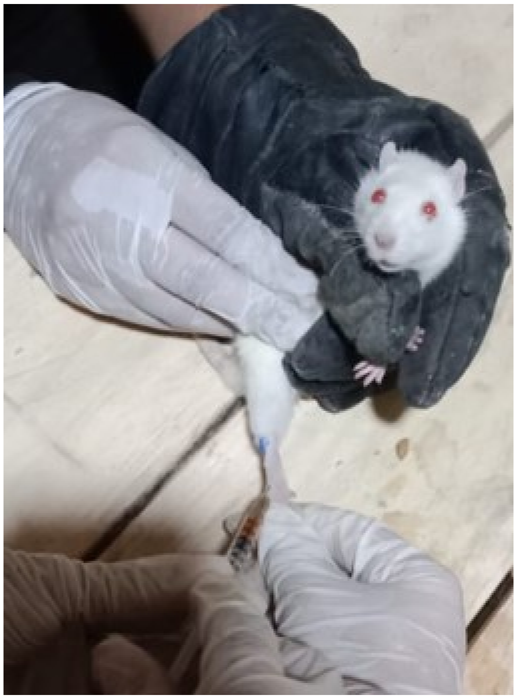

Figure S7. Paw oedema was induced by using carrageenan.

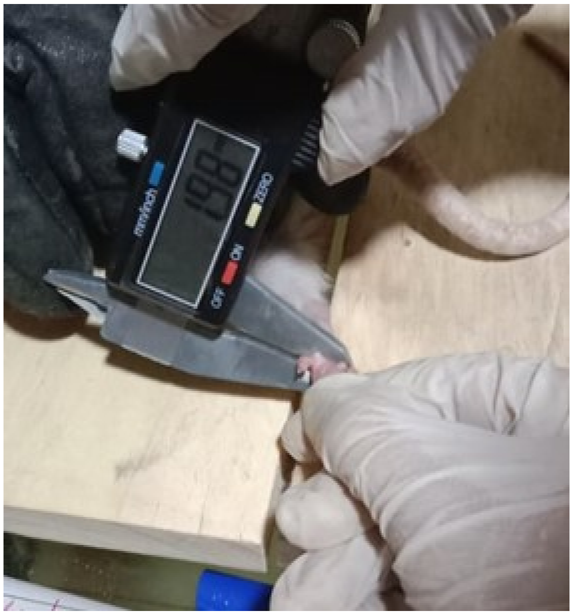

Figure S8. Paw oedema after 3 hours of carrageenan injection.

In vivo Antipyretic Activity

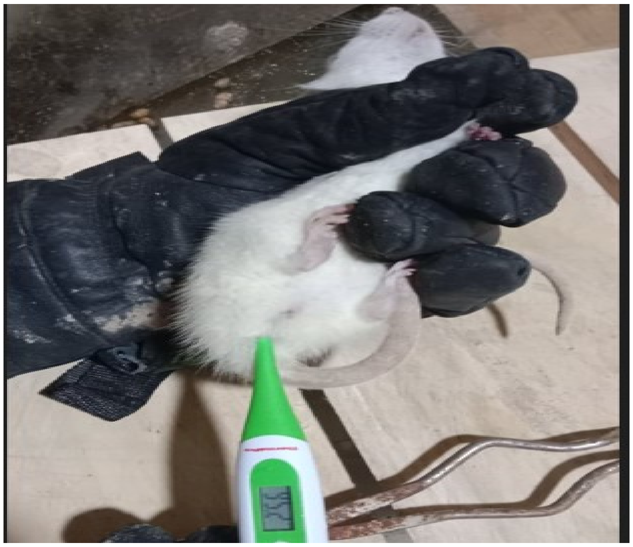

Figure S9. average rectal temperature of the animal group at the beginning.

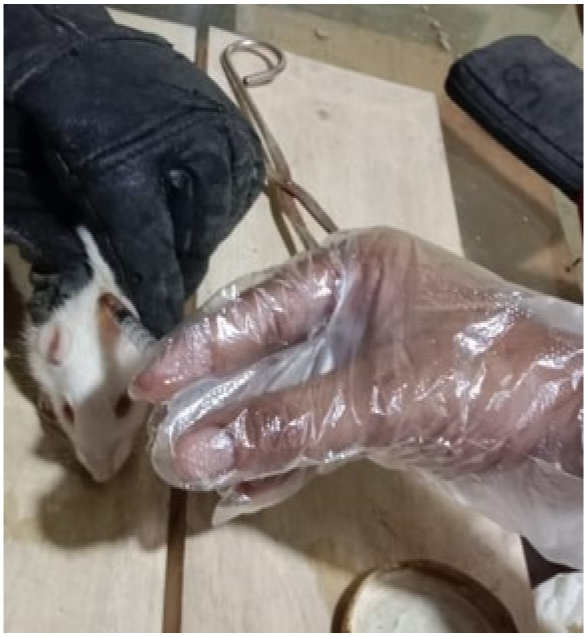

Figure S10. Subcutaneous injection of yeast.

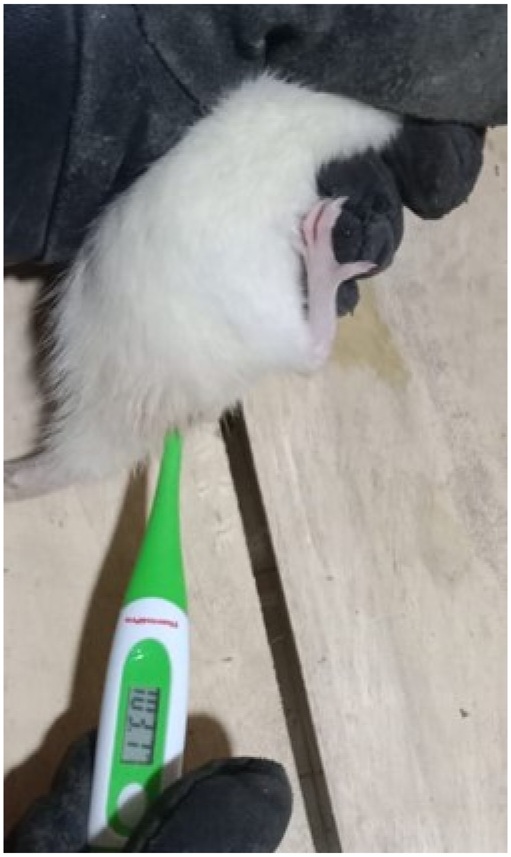

Figure S11. increase in rectal temperature, which after yeast injection at 101F°

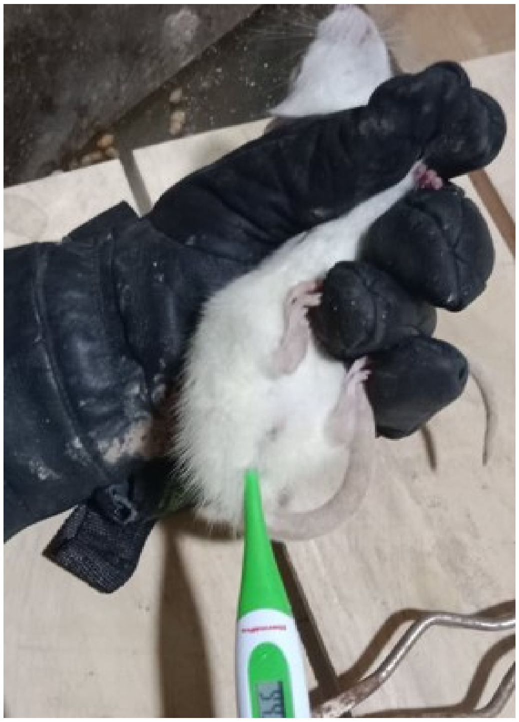

Figure S12. Temperature dropped to 99.1F° after treatment with mushroom fraction.

Table S1. HPLC analysis of Phenolics and flavonoids Compounds (ppm) of active fractions of methanolic extract of *Lentinula edodes*.

Sr Num	Fractions	Phytochemicals	Retention time (min)	Area (%)	Concentration (ppm)
**1**	Chloroform	Quercetin<br>Caffeic acid<br>Vanillic acid<br>Benzoic acid<br>Synergic acid<br>p-coumaric acid<br>Cinnamic acid<br>Sinapic acid	3.093<br>12.680<br>13.013<br>14.853<br>16.527<br>17.933<br>24.973<br>26.227	0.1<br>0.7<br>1.6<br>4.1<br>1.8<br>3.9<br>7.4<br>3.6	0.4183<br>2.00<br>6.0741<br>26.6569<br>2.7873<br>3.0502<br>15.7983<br>2.8679
**2**	Hexane	Quercetin<br>Gallic acid	3.027<br>4.527	6.00<br>3.9	36.4325<br>16.3091
**3**	Ethyl acetate	Quercetin<br>Gallic acid<br>Benzoic acid	2.973<br>4.100<br>14.893	0.3<br>4.7<br>6.2	8.556<br>87.8679<br>345.871

Table S2. FTIR. Functional groups with Wave-number (cm-1).

Sr No.	Wave Number cm^{-1}	Functional group
**1**	1738.49cm^{-1}	Aldehyde (C=O)
**2**	1710.55cm^{-1}	Conjugated aldehyde (C=O)
**3**	2855.21cm^{-1}-2960.03cm-1	Alkane(C-H)
**4**	2925. 88cm^{-1}	Amine salt(N-H)
**5**	1727.72cm-1	α,β-unsaturated ester (C=O)
**6**	1741.70cm-1	Esters(C=O)
**7**	3363.67cm-1	Alcohols(O-H)

Table S3. In silico analysis of Antioxidant activity

Compounds	Pubchem ID	Melatonin	Myeloperoxidase	NADPH oxidase
4H-Furo[3,2-b]pyrrole-5-carboxylic acid	7141881	-6.0419	-7.3	-6.2228
9,10-Di(methylthio)hexadecanoic acid, methyl ester	612361	-6.5500	-6.0080	-5.3621
9,12-Octadecadienoic acid (Z,Z)-, methyl ester	5284421	-6.2742	-5.3960	-5.1105
N-(4-Isopropylbenzyl)-3-phenylpropionamide	563207	-5.7379	-5.2644	-4.6414
trans-3-Ethoxy-b-methyl-b-nitrostyrene	45094110	-5.2784	-4.1814	-4.5791
4-Aminobenzoic acid, 2TMS derivative	530153	-5.7511	-4.4635	-4.9182
Butanoic acid, octyl ester	61030	-5.6444	-4.2454	-4.6065
3-Trifluoromethyl-7-phenothiazone	620167	-4.9821	-5.7158	-4.2735
4-Dehydroxy-N-(4,5-methylenedioxy-2-nitrobenzylidene)tyramine	610062	-5.2003	-4.9419	-4.8433
Pyrido[1,2-a]pyrimidin-4(5H)-one, 9-hydroxy-2-methyl-8-nitro	534111	-4.8542	-5.9472	-4.1882

1,4-4-Bis(trimethylsilyl)benzene	25771	-5.2138	-4.1829	-4.5030
5-Methyl-6-phenyltetrahydro-1,3-oxazine-2-thione	5370596	-4.6471	-4.4761	-4.1145
trans-2,3-Methylenedioxy-b-methyl-b-nitrostyrene	11694143	-4.6252	-4.2643	-4.1173
4H-Furo[3,2-b]pyrrole-5-carboxylic acid, 4-(2-oxopropyl)-	6537090	-4.2247	-3.9937	-4.0474
Propanamide	6578	-3.6733		-3.2991

Table S4. *In vivo* Anti-inflammatory effects of *Lentinula edodes* selected fractions of methanolic extracts on carrageenan-induced inflammation.

**Sample fraction**	**Dose mg/kg body weight**	**Change in Paw thickness(mm) 0h**	**1h**	**2h**	**3h**	**4h**
Chloroform	**200**	1.41±0.04<br>12.5%	1.52±0.03<br>20.10%	1.62±0.04<br>26.81%	1.71±0.04<br>38.92%	1.23±0.03<br>57.29%
	**300**	1.31±0.02<br>23.07%	1.41±0.06<br>25.39%	1.52±0.03<br>28.09%	1.63±0.04<br>42.5%	1.19±0.03<br>60.99%
	**400**	1.13±0.05<br>34.9%	1.31±0.03<br>37.61%	1.42±0.04<br>41.25%	1.52±0.03<br>46.07%	1.18±0.06<br>63.21%
Ethyl acetate	**200**	1.26±0.04<br>10%	1.35±0.03<br>14.55%	1.41±0.04<br>16.56%	1.51±0.04<br>20.10%	1.2±0.03<br>50%
	**300**	1.25±0.02<br>15.5%	1.31±0.06<br>18.63%	1.42±0.03<br>21.22%	1.52±0.04<br>23.73%	1.15±0.03<br>56%
	**400**	1.21±0.05<br>17.6%	1.34±0.03<br>20.23%	1.41±0.04<br>25.39%	1.51±0.03<br>28.09%	1.13±0.06<br>57%
Hexane	**200**	1.19±0.04<br>8.46%	1.28±0.03<br>11.72%	1.38±0.04<br>14.28%	1.48±0.04<br>23.31%	1.31±0.03<br>37%
	**300**	1.15±0.02<br>16.66%	1.21±0.06<br>19.8%	1.31±0.03<br>23.39%	1.42±0.04<br>28.28%	1.23±0.03<br>42%
	**400**	1.13±0.05<br>18.70%	1.21±0.03<br>24.52%	1.32±0.04<br>27.47	1.43±0.03<br>31.90%	1.14±0.06<br>47%
Control group	**200**	1.38±0.04<br>30%	1.47±0.03<br>1%	1.57±0.04<br>2%	1.65±0.04<br>15%	1.64±0.03<br>0%
	**300**	1.41±0.02<br>40%	1.52±0.06<br>2%	1.63±0.03<br>3%	1.72±0.04<br>1%	1.73±0.03<br>0%
	**400**	1.25±0.05<br>3%	1.35±0.03<br>1%	1.45±0.04<br>3%	1.52±0.03<br>1%	1.53±0.06<br>0%
Diclofenac Sodium Standard	**200**	1.12±0.03<br>31	1.31±0.04<br>45%	1.41±0.02<br>58%	1.53±0.02<br>61%	1.2±0.02<br>70%
	**300**	1.14±0.02<br>35%	1.21±0.06<br>47%	1.32±0.03<br>56%	1.41±0.04<br>67%	1.13±0.03<br>75%
	**400**	1.25±0.05<br>38%	1.35±0.03<br>45%	1.45±0.04<br>57%	1.56±0.03<br>66%	1.21±0.06<br>76%
